# MicroRNA-582-5p inhibits the progression of gastric cancer cells and their resistance to oxaliplatin by suppressing ATG7 expression

**DOI:** 10.3389/fonc.2024.1481266

**Published:** 2024-10-11

**Authors:** Yu Chang, Yaqin Hao, Yani Su, Jin Guo, Yu Liu, Ruixue Sun, Bei Feng, Junwei Ma, Yunfeng Hu

**Affiliations:** ^1^ Department of Radiation Oncology, The First Affiliated Hospital of Yan’an University, Yan’an, Shanxi, China; ^2^ Department of Clinical Laboratory Medicine, Cancer Hospital of Shantou University Medical College, Shantou, China; ^3^ Department of Oncology, The First Affiliated Hospital of Yan’an University, Yan’an, Shanxi, China

**Keywords:** gastric cancer, microRNA, chemotherapy resistance, ATG7, autophagy

## Abstract

**Background:**

Gastric cancer (GC) is one of the most common malignant tumors of the digestive tract worldwide. Both environmental and genetic factors contribute to the occurrence and development of GC. Surgery and chemotherapy are the main treatment modalities for gastric cancer; however, some patients show insensitivity to chemotherapeutic agents. Chemotherapy resistance is one of the primary reasons for poor treatment outcomes and the high likelihood of recurrence and metastasis in gastric cancer patients. Numerous studies have confirmed a correlation between the dysregulation of microRNA expression and the development of various malignant tumors, as well as their resistance to chemotherapeutic agents. However, the role of microRNA-582-3p in gastric cancer cells and its mechanism in the resistance of gastric cancer cells to oxaliplatin have not been studied.

**Methods:**

We first used q-PCR, CCK8, transwell, and scratch assays to validate the expression of microRNA-582-3p in gastric cancer tissues and cells, while also analyzing the relationship between its expression levels and the clinical pathological data of patients. Additionally, we further confirmed the impact of microRNA-582-3p on gastric cancer cell progression and oxaliplatin resistance through knockdown and overexpression experiments. Subsequently, to explore the specific mechanisms of microRNA-582-3p in gastric cancer, we verified the downstream target of microRNA-582-3p, ATG7, using dual-luciferase reporter assays and examined the effect of ATG7 on gastric cancer cell functions. Moreover, we conducted rescue experiments to further validate the interaction between microRNA-582-3p and ATG7.

**Results:**

Our experimental results confirmed that microRNA-582-3p is lowly expressed in gastric cancer tissues and cells, and the expression level of miR-582-5p is correlated with the T stage of patients, while showing no correlation with the patients' gender, age, tumor size, degree of differentiation, or N stage. Additionally, we found that microRNA-582-3p functions as a tumor suppressor in gastric cancer cells, as its overexpression inhibits the biological functions of gastric cancer cells and increases their sensitivity to oxaliplatin. Furthermore, we identified binding sites between microRNA-582-3p and the autophagy-related gene ATG7, observing that knockdown of microRNA-582-3p increases ATG7 expression, while its overexpression reduces ATG7 levels. Moreover, ATG7 is overexpressed in gastric cancer cells; knockdown of ATG7 inhibits the biological functions of gastric cancer cells and increases their sensitivity to oxaliplatin, whereas overexpression of ATG7 reverses the inhibitory effect of miR-582-5p on gastric cancer.

**Conclusion:**

Our study confirms that microRNA-582-3p acts as a tumor suppressor in gastric cancer cells, and its role may be mediated through the regulation of ATG7 expression levels. MicroRNA-582-3p may serve as a potential target for gastric cancer treatment and a predictive biomarker.

## 1 Introduction

Gastric cancer (GC) is one of the most common malignant tumors globally and a leading cause of cancer-related deaths (1). Approximately one million people worldwide are diagnosed with gastric cancer annually, resulting in about 738,000 deaths (2). Current primary treatments for gastric cancer patients include chemotherapy, targeted therapy, and immunotherapy, with chemotherapy being the main approach for postoperative and advanced-stage patients (3). Currently, oxaliplatin (L-OHP) is predominantly used in chemotherapy regimens for gastric cancer patients in clinical practice (4).

Platinum-based chemotherapy drugs are currently the most widely used chemotherapeutic agents in clinical practice, and they exhibit significant anticancer efficacy (5). Oxaliplatin is a third-generation platinum-based chemotherapy drug that primarily functions by forming complexes with DNA *in vivo*, disrupting DNA replication, and inhibiting cell division in tumor cells to achieve its anticancer effects (6). Oxaliplatin is also employed in adjuvant chemotherapy regimens for gastric cancer, and developing resistance to oxaliplatin is a major reason for poor treatment outcomes, worse prognosis, and recurrence and metastasis in gastric cancer patients (7). Therefore, studying the molecular mechanisms influencing oxaliplatin resistance in gastric cancer patients is crucial.

Autophagy is an evolutionarily conserved catabolic process whereby damaged or defective cellular components are degraded by lysosomes to provide energy for cellular metabolism. It plays a crucial role in maintaining cellular homeostasis and regulating growth processes (8). The initiation and formation of autophagosomes are mediated by autophagy-related genes (ATGs), with over 30 ATGs currently identified (9). Research has shown that autophagy plays a dual role in gastric cancer regarding chemotherapy drug resistance,it can both promote resistance of gastric cancer cells to chemotherapy drugs and enhance sensitivity to these drugs (10). ATG7 is a crucial effector enzyme in autophagy, damage to ATG7 can lead to defects in autophagy within tissues and cells (11). ATG7 also plays a role in the resistance of malignant tumors to chemotherapy drugs. for instance, studies have demonstrated that MiR-7-5p enhances bladder cancer resistance to cisplatin by downregulating ATG7 (12). CD13 induces autophagy via the P38/Hsp27/CREB/ATG7 pathway to promote resistance of liver cancer cells to chemotherapy drugs (13). Thus, ATG7 plays a significant role in cancer resistance to chemotherapy drugs.

MicroRNAs are a class of small non-coding RNAs approximately 18-22 nucleotides in length, primarily exerting their effects by binding to the 3’UTR of target messenger RNAs (mRNAs) to inhibit the expression of associated proteins (14, 15). Extensive research has demonstrated that MicroRNAs play crucial roles in the pathogenesis of human cancers. For example, Mir-375 has been identified as a tumor suppressor in various malignant tumor cells, regulating processes such as proliferation, migration, invasion, and apoptosis by binding to its target genes (16). Mir-7 also plays significant roles in multiple cancers, participating not only in signaling pathway regulation but also serving as a biomarker and modulating the expression of target proteins (17). Current studies have revealed that Mir-582-5p has regulatory effects on the biological functions of malignancies such as bladder cancer (18), colorectal cancer (19), prostate cancer (20), and non-small cell lung cancer (21). However, there is no reported research on whether Mir-582-5p influences the malignant biological functions of gastric cancer cells and their resistance to oxaliplatin through the regulation of autophagy.

In this study, we found that Micro-582-5p is downregulated in gastric cancer tissues and cells. Overexpression of Micro-582-5p inhibited the proliferation, migration, and invasion of gastric cancer cells, and increased their sensitivity to oxaliplatin. Additionally, we observed high expression of ATG7 in gastric cancer tissues and cells. Knockdown of ATG7 suppressed the malignant biological functions of gastric cancer cells and enhanced their sensitivity to oxaliplatin. Subsequently, co-overexpression of Micro-582-5p and ATG7 resulted in enhanced biological functions compared to overexpression of Micro-582-5p alone, but decreased sensitivity to oxaliplatin. Therefore, we suggest that Micro-582-5p may enhance resistance of gastric cancer cells to oxaliplatin by inhibiting ATG7.

## 2 Materials and methods

### 2.1 Source of patient tissue samples

Fresh gastric cancer tissue samples were obtained from patients diagnosed with gastric cancer at the Affiliated Hospital of Yan’an University. Immediately following surgical resection, pathological samples were collected and promptly preserved in liquid nitrogen. Additionally, paraffin-embedded tissue samples from gastric cancer patients with confirmed pathological diagnoses were also acquired from the same hospital.

### 2.2 Cell lines and culture

Human gastric cancer cell lines HGC 27,MKN 28, and AGS, as well as the human normal gastric mucosal cell line GES-1, were provided by the Medical Translational Center of Yan’an University School of Medicine. HCG27 and MKN28 cells were cultured in RPMI-1640 medium supplemented with 10% fetal bovine serum (FBS). AGS and GES cells were cultured in DMEM medium supplemented with 10% FBS. All cells were maintained in a cell culture incubator at 37°C with 5% CO2.

### 2.3 qRT-PCR

Total RNA was extracted from gastric cancer cell lines and normal/tumor paired tissues using TRIzol reagent (AG) according to the manufacturer’s instructions. RNA was reverse transcribed into cDNA using the RT-PCR reverse transcription kit (Aikerui Biosciences, China, AG11711). qRT-PCR was performed using SYBR Green Pro Taq HS premix qPCR kit (Aikerui Biosciences, China, AG11701) on a Roche Cobas Z480, with U6 and GAPDH as internal references. Relative expression levels were calculated using the 2^-ΔΔCT method. Primer sequences are listed in **Supplementary Table 1**.

### 2.4 Cell transfection

MiR-582-5p mimic, mimic control (mimic-NC), MiR-582-5p inhibitor,(inhibitor-NC) small interfering RNA targeting ATG7 (si-ATG7), and its negative control (si-NC) were purchased from Aikerui Biosciences. Transfection was performed using jetPRIME^®^ transfection reagent (Polyplus) following the manufacturer’s protocol. Cells were collected for subsequent studies 48 hours post-transfection. Sequences for small interfering RNA, MiR-582-5p mimic, MiR-582-5p, and mimic control (miR-NC) are detailed in **Supplementary Table 1**.

### 2.5 CCK-8 assay

Cells (2 × 103) were seeded in 96-well plates and cultured until specific time points. Each well was treated with 10 μL CCK-8 reagent and incubated for 2 hours in the culture incubator. Optical density (OD) at 450 nm was measured using a microplate reader.

### 2.6 Scratch assay

Cells in logarithmic growth phase were evenly seeded in 6-well plates (approximately 4 × 10^5^ cells per well) and cultured in the incubator. After 24 hours, cells were transfected according to the Polyplus transfection reagent instructions, with culture medium without antibiotics added. After 6 hours of transfection, scratches were made using a 100 µl pipette tip. Images were taken under a microscope as 0-hour controls, and wound healing was observed and quantified at 24 and 48 hours post-scratch. Cell migration rate was calculated using the formula: Cell migration rate = [(initial blank area - blank area at specific time point)/initial blank area] × 100%.

### 2.7 Transwell assay

Transwell chambers were used to evaluate the migration and invasion capabilities of cancer cells. Transfected gastric cancer cells were seeded in the chambers. For invasion assays, matrix gel was coated on the upper chamber, while for migration assays, cells were directly seeded on the upper chamber. The lower chamber was filled with medium containing 20% FBS. After 48 hours of incubation, cells were fixed with 4% paraformaldehyde for 30 minutes and stained with 0.25% crystal violet.

### 2.8 Dual-luciferase reporter assay

The wild-type (WT) fragment of ATG7 3′-UTR containing the binding sequence of miR-582-5p was cloned into pmirGLO vector to construct ATG7-WT. Mutant (MUT) fragment (ATG7-MUT) with mutated binding sequence was also cloned into pmirGLO vector. Gastric cancer cells were co-transfected with ATG7-WT or ATG7-MUT and miR-582-5p mimic or miR-NC using Polyplus reagent. After 48 hours, firefly and Renilla luciferase activities were measured using the dual-luciferase reporter gene assay system.

### 2.9 Western blotting

Total protein from cells and tissues was extracted using RIPA buffer. Protein samples were separated by 10% SDS-PAGE and transferred onto PVDF membranes. Membranes were blocked with 5% skim milk at room temperature for 2 hours, followed by overnight incubation with primary antibodies (GAPDH, P62, ATG7, Beclin1) at 4°C. After washing, membranes were incubated with HRP-conjugated secondary antibodies (goat anti-rabbit IgG) at room temperature for 2 hours. Protein bands were visualized using ECL exposure strips.

### 2.10 Statistical Analysis

All experiments were repeated three times, and data are presented as mean ± SD (standard deviation). Student’s t-test was used for statistical analysis. P< 0.05 was assumed to be statistically significant.

## 3 Results

### 3.1 The expression and clinical significance of miR-582-5p in gastric cancer

**Figure 1:**
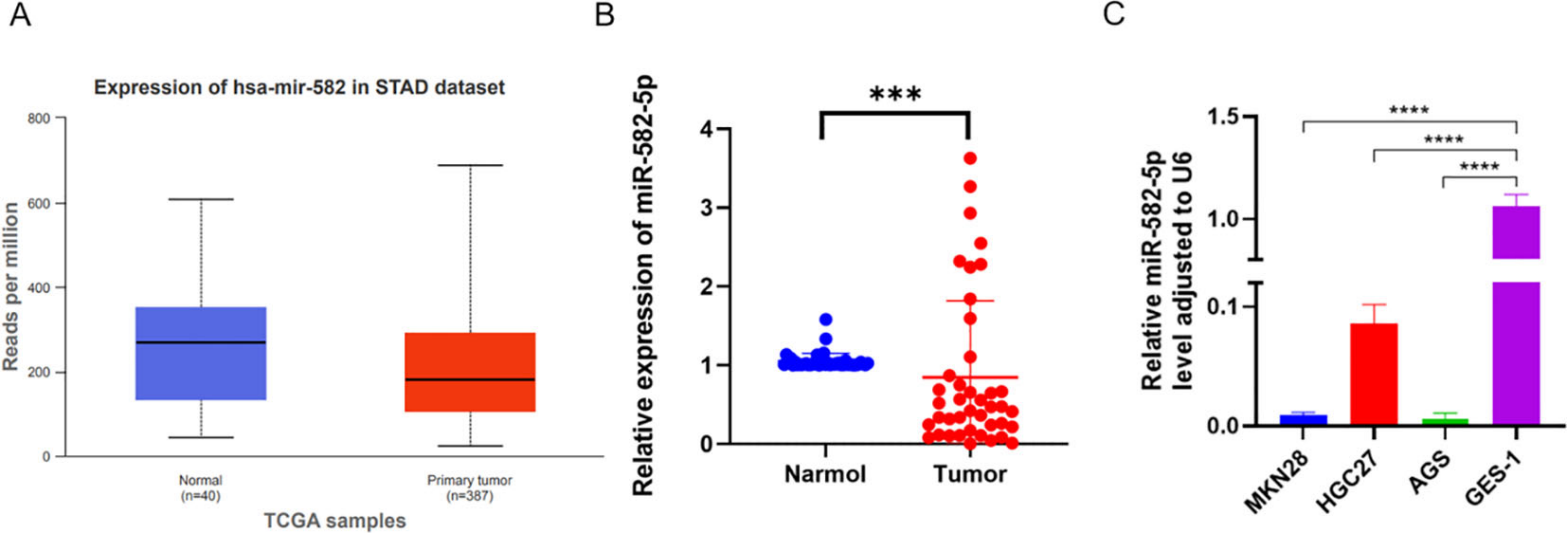
Expression of MiR-582-5p in gastric cancer and its association with patient prognosis. **(A)** Expression of MiR-582-5p in gastric cancer tissues from UALCAN database. **(B)** Expression of MiR-582-5p in GC tissues and normal tissues detected by qRT-PCR. **(C)** Expression of MiR-582-5p in gastric cancer cells and GES-1 cells detected by qRT-PCR. (***P < 0.001).

To investigate the expression of miR-582-5p in gastric cancer and its relationship with clinical pathological parameters, we first identified low expression of miR-582-5p in gastric cancer using the UALCAN database (https://ualcan.path.uab.edu/) (**Figure 1A**). We further examined miR-582-5p expression in 41 pairs of gastric cancer and normal tissue samples by qPCR, confirming low expression in gastric cancer tissues (**Figure 1B**). Additionally, we assessed miR-582-5p expression in gastric cancer cell lines and normal gastric epithelial cells, demonstrating consistently low expression in gastric cancer cell lines (**Figure 1C**). Analysis of the correlation between miR-582-5p expression and clinical pathological parameters in patients indicated a significant association with T stage, while no correlation was observed with gender, age, tumor size, differentiation grade, or N stage (**Table 1**).

**Table 1:** Correlation between MiR-582-5p expression and clinical pathological parameters in patients.

Clinical features	Number of cases n=41	MiR-582-5p		*P*
		High (n=11)	Low(n=30)	
Gender				
Male	35	10	25	1.00
Female	6	1	5	
Age				
≤60 years old	14	5	9	0.463
>60 years old	27	6	21	
Tumor size				
≤5cm	23	6	17	1.00
>5cm	18	5	13	
Differentiation grade				
Poorly/Moderately differentiated	34	10	24	0.651
Moderately/Well differentiated	7	1	6	
T stage				
T1+T2	5	4	1	0.014^*^
T3+T4	36	7	29	
N stage				
N0	11	4	7	0.445
N1-3	30	7	23	

(*p<0.05).

### 3.2 Overexpression of MiR-582-5p inhibits proliferation, migration, invasion, and resistance to oxaliplatin in gastric cancer cells

**Figure 2:**
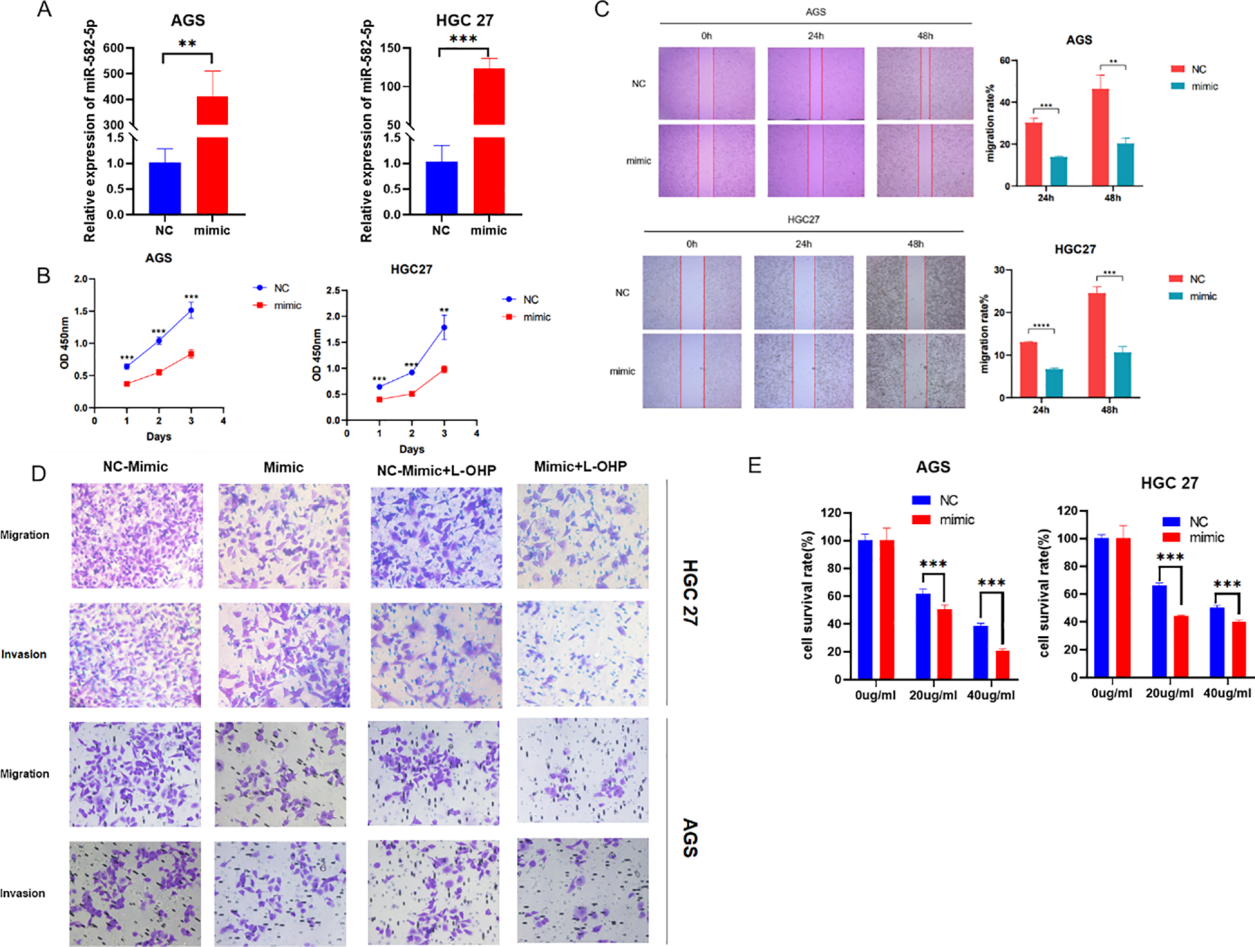
Overexpression of MiR-582-5p Inhibits Proliferation, Migration, Invasion, and Resistance to Oxaliplatin in Gastric Cancer Cells. **(A)** Transfection efficiency was verified by q-PCR; **(B)** Overexpression of MiR-582-5p inhibited the proliferation ability of gastric cancer cells; **(C)** Overexpression of MiR-582-5p suppressed the migration ability of gastric cancer cells; **(D)** Overexpression of MiR-582-5p significantly inhibited the migration and invasion capabilities of gastric cancer cells; **(E)** Overexpression of MiR-582-5p increased the sensitivity of gastric cancer cells to oxaliplatin. (**P < 0.01, ***P < 0.001).

To explore the biological function of MiR-582-5p in gastric cancer cells, we constructed a MiR-582-5p overexpression gastric cancer cell line. We verified the transfection efficiency using q-PCR (**Figure 2A**). Next, we assessed the effect of MiR-582-5p overexpression on the proliferation ability of gastric cancer cells using the CCK8 assay, which showed that overexpression of MiR-582-5p significantly inhibited the proliferation of gastric cancer cells (**Figure 2B**). Meanwhile, wound healing assays demonstrated that overexpression of MiR-582-5p suppressed the invasion and migration capabilities of gastric cancer cell lines (**Figure 2C**). These experimental results confirm that MiR-582-5p acts as a tumor suppressor gene, effectively inhibiting the malignant biological functions of gastric cancer cells. Subsequently, we investigated whether MiR-582-5p affects the sensitivity of gastric cancer cells to oxaliplatin. We demonstrated through CCK8 and transwell assays that MiR-582-5p can enhance the sensitivity of gastric cancer cells to oxaliplatin (**Figures 2D, E**).

### 3.3 Knockdown of MiR-582-5p promotes proliferation, migration, invasion, and resistance to oxaliplatin in gastric cancer cells

**Figure 3:**
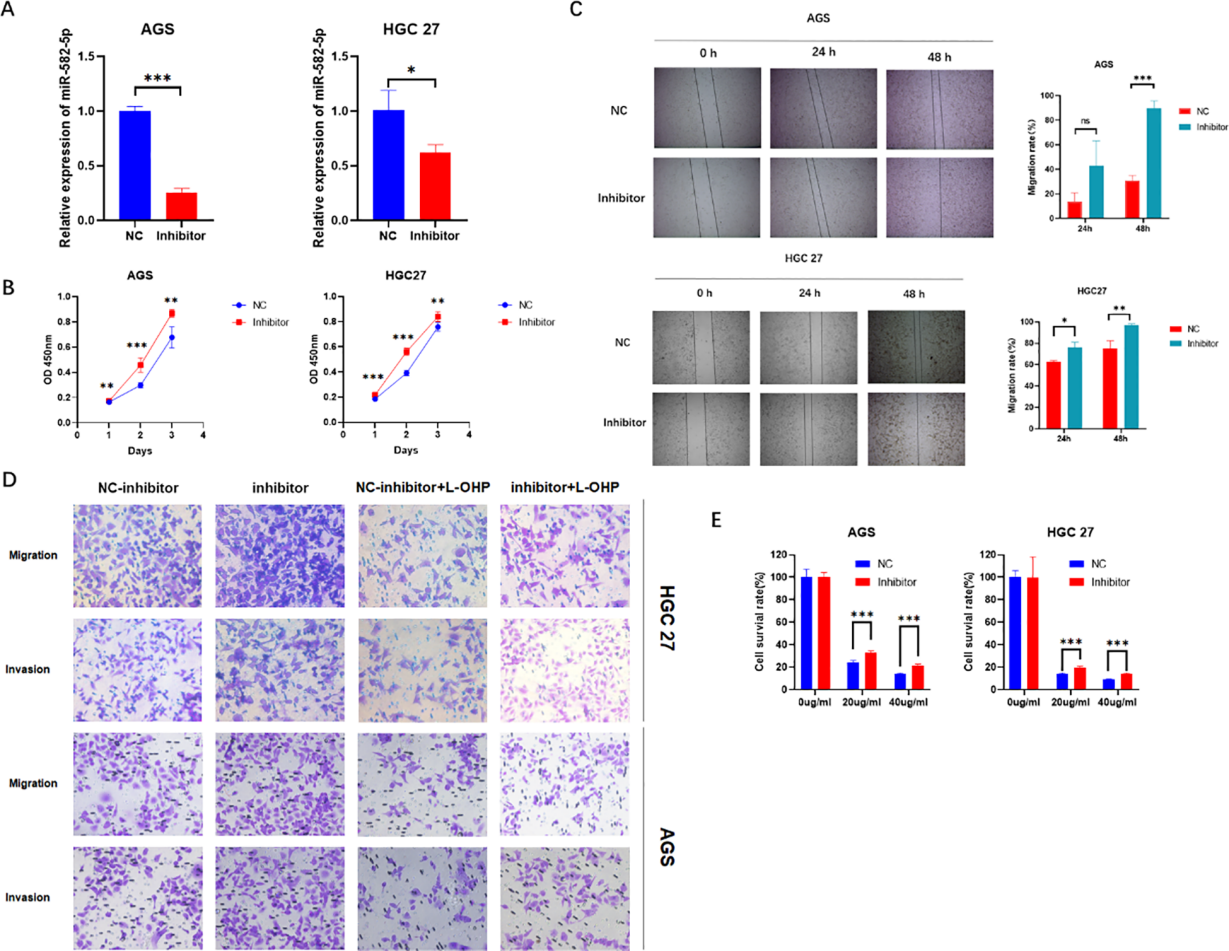
Knockdown of MiR-582-5p Promotes Proliferation, Migration, Invasion, and Resistance to Oxaliplatin in Gastric Cancer Cells **(A)** Transfection efficiency was verified by q-PCR; **(B)** Knockdown of MiR-582-5p promoted the proliferation ability of gastric cancer cells; **(C)** Knockdown of MiR-582-5p enhanced the migration ability of gastric cancer cells; **(D)** Knockdown of MiR-582-5p significantly promoted the migration and invasion capabilities of gastric cancer cells; **(E)** Knockdown of MiR-582-5p reduced the sensitivity of gastric cancer cells to oxaliplatin. (ns P > 0.05, *P < 0.05, **P < 0.01, ***P < 0.001).

To further confirm the biological function of MiR-582-5p in gastric cancer cells, we constructed a MiR-582-5p knockdown gastric cancer cell line. The transfection efficiency was verified by q-PCR (**Figure 3A**). The CCK8 assay assessed the effect of MiR-582-5p knockdown on the proliferation ability of gastric cancer cells, revealing that knockdown of MiR-582-5p significantly promoted the proliferation of gastric cancer cells (**Figure 3B**). Meanwhile, wound healing assays demonstrated that knockdown of MiR-582-5p enhanced the invasion and migration capabilities of gastric cancer cells (**Figure 3C**). Next, we investigated the effect of MiR-582-5p knockdown on the sensitivity of gastric cancer cells to oxaliplatin. CCK8 and transwell assays confirmed that knockdown of MiR-582-5p can reduce the sensitivity of gastric cancer cells to oxaliplatin (**Figures 3D, E**).

### 3.4 ATG7 is a target of MiR-582-5p

**Figure 4:**
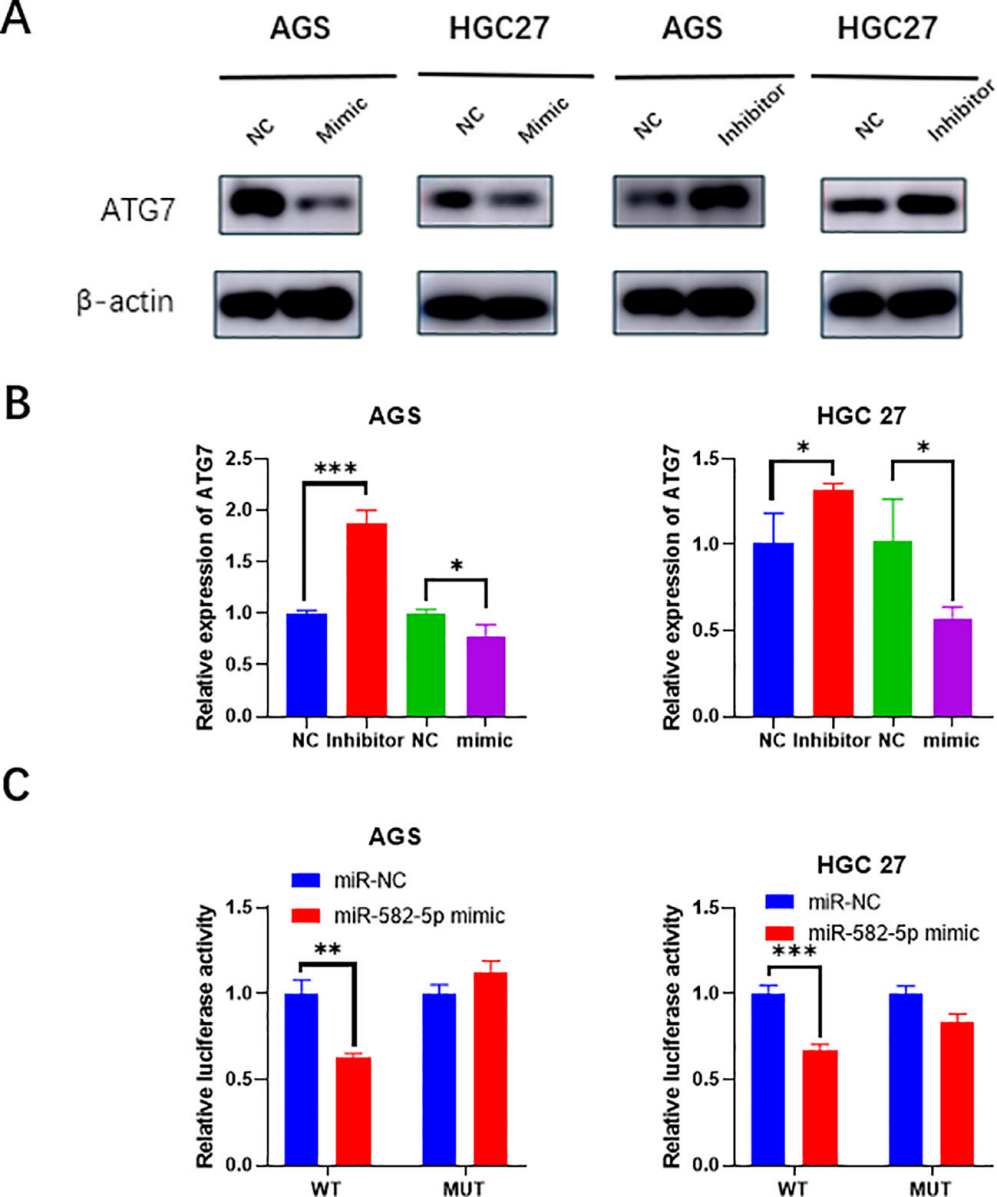
ATG7 is a target of MiR-582-5p. **(A)** Western blotting demonstrated changes in ATG7 protein expression following MiR-582-5p overexpression and knockdown. **(B)** q-PCR showed changes in ATG7 RNA expression after MiR-582-5p overexpression and knockdown. **(C)** Dual-luciferase reporter assay confirmed the interaction between MiR-582-5p and ATG7. (*P < 0.05, **P < 0.01, ***P < 0.001).

To further investigate the mechanism by which MiR-582-5p affects gastric cancer cell biological functions and sensitivity to oxaliplatin, we assessed the expression of ATG7 at both the RNA and protein levels following MiR-582-5p overexpression and knockdown using q-PCR and western blotting. The results showed that overexpression of MiR-582-5p led to low expression of ATG7, while knockdown of MiR-582-5p resulted in high expression of ATG7 (**Figures 4A, B**). This suggests that ATG7 might be a potential downstream target of MiR-582-5p and that MiR-582-5p could exert its effects by targeting ATG7. To confirm this, we performed a dual-luciferase reporter assay to verify the binding sites. The results indicated that MiR-582-5p has binding sites in the 3′-UTR of ATG7 mRNA (**Figure 4C**).

### 3.5 Expression and clinical significance of ATG7 in gastric cancer

**Figure 5:**
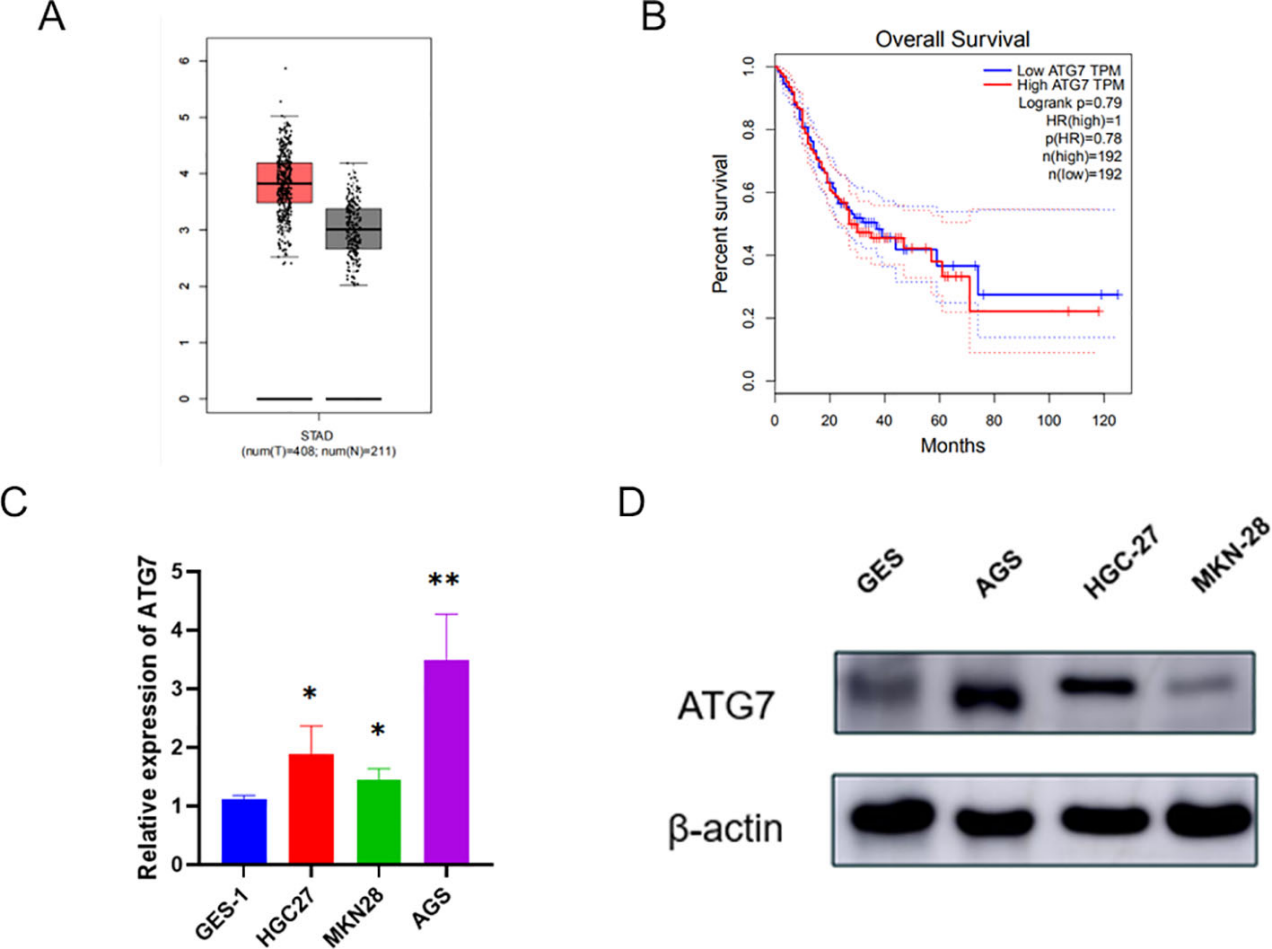
Expression and Clinical Significance of ATG7 in Gastric Cancer **(A)** Expression of ATG7 in gastric cancer. **(B)** Relationship between ATG7 expression and prognosis of gastric cancer patients. **(C)** Expression of ATG7 RNA in gastric cancer cells and normal cells detected by qRT-PCR. **(D)** Expression of ATG7 protein in gastric cancer cells and normal cells detected by Western blotting. (*P < 0.05, **P < 0.01).

Next, we further explored the impact of ATG7 on the biological functions of gastric cancer cell lines. First, using the GEPIA database, we found that ATG7 is highly expressed in gastric cancer (**Figure 5A**), and that patients with high ATG7 expression have a lower overall survival (OS) compared to those with low expression (**Figure 5B**). We used the normal gastric mucosal epithelial cell line GES-1 as the control group and selected three gastric cancer cell lines (HGC27, MKN28, AGS) as the experimental group. Total RNA and protein were extracted from each group of cells, and the expression levels of ATG7 were measured using RT-qPCR and western blotting. The results showed that ATG7 expression levels were significantly upregulated in the three gastric cancer cell lines (HGC27, MKN28, AGS) compared to the normal gastric mucosal epithelial cell line GES-1 (**Figures 5C**, **D**) (*p<0.05, **p<0.01, ***p<0.001).

### 3.6 Knockdown of ATG7 suppresses proliferation, invasion, migration, and oxaliplatin resistance of gastric cancer cells

**Figure 6:**
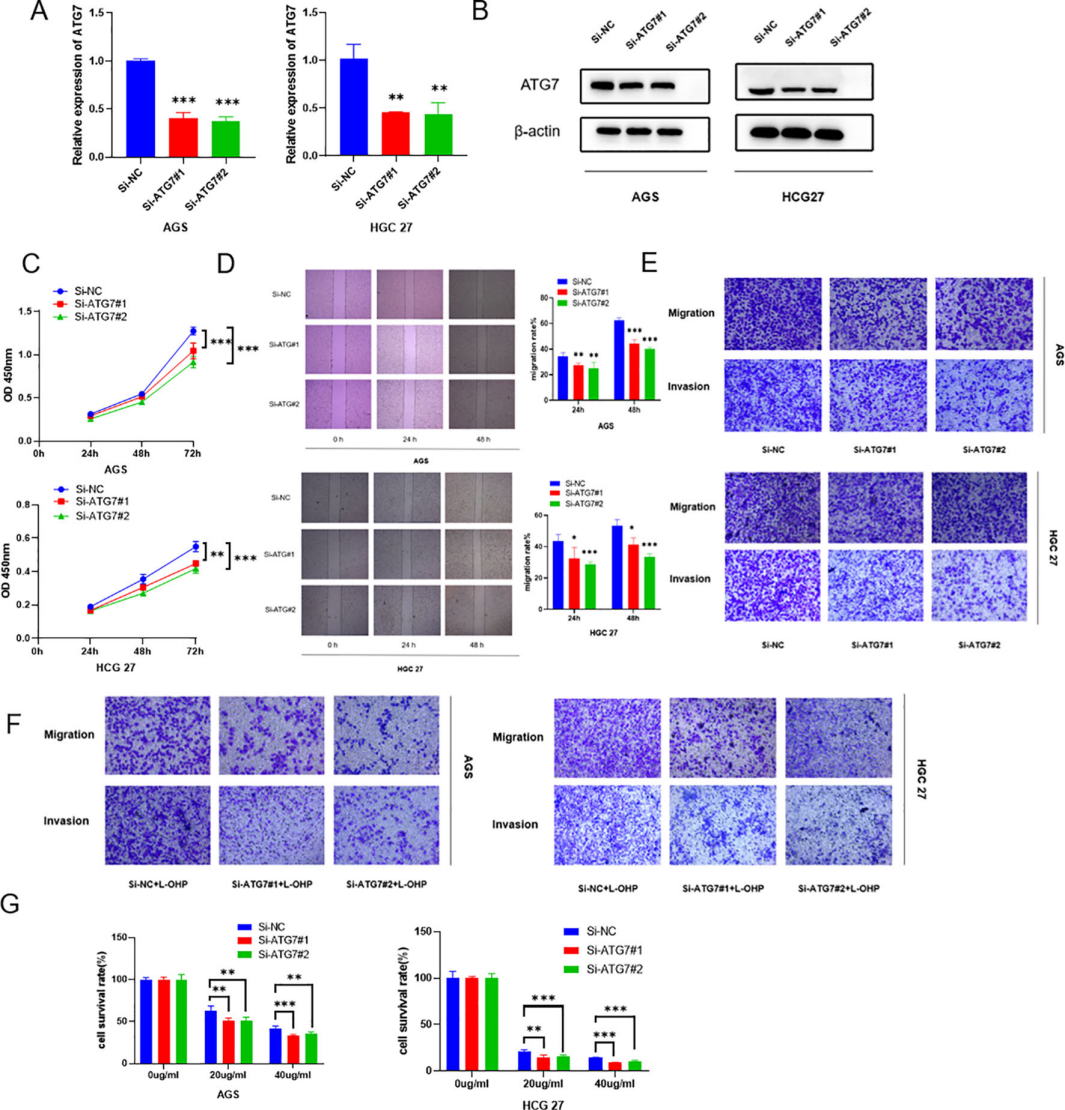
Knockdown of ATG7 suppresses proliferation, invasion, migration, and oxaliplatin resistance of gastric cancer cells **(A)** qRT-PCR analysis showing reduced RNA expression levels of ATG7 after ATG7 knockdown. **(B)** Western blotting analysis demonstrating decreased protein expression levels of ATG7 after ATG7 knockdown. **(C)** CCK8 assay showing the impact of ATG7 knockdown on proliferation capacity of gastric cancer cells. **(D)** Scratch assay demonstrating the effect of ATG7 knockdown on migration capacity of gastric cancer cells. **(E)** Transwell assay showing the effect of ATG7 knockdown on invasion and migration of gastric cancer cells. **(F)** Transwell assay evaluating the effect of ATG7 knockdown on chemotherapeutic drug resistance of gastric cancer cells. **(G)** CCK8 assay evaluating the effect of ATG7 knockdown on sensitivity of gastric cancer cells to oxaliplatin. (*P < 0.05, **P < 0.01, ***P < 0.001).

By examining ATG7 expression in gastric cancer cells, we observed high levels of ATG7 expression. To investigate whether its overexpression affects the biological functions of gastric cancer, we designed two siRNAs targeting ATG7 and established transiently transfected gastric cancer cell lines with reduced ATG7 expression. We validated the knockdown efficiency through RT-qPCR and Western blotting (**Figures 6A**, **B**). Functional assays were performed on HGC27, MKN28, and AGS cells with transient ATG7 knockdown. The results showed that compared to the negative control (NC) group, proliferation capacity was decreased in HGC27 and AGS cells in the CCK8 proliferation assay (**Figure 6C**). Scratch and Transwell assays demonstrated reduced migration and invasion capabilities of cells following ATG7 knockdown (**Figures 6D**, **E**). Additionally, using Transwell and CCK8 assays, we evaluated the impact of ATG7 knockdown on gastric cancer cell resistance to oxaliplatin, revealing suppressed proliferation, invasion, migration, and oxaliplatin resistance after ATG7 knockdown (**Figures 6F, G**) (*P < 0.05, **P < 0.01, ***P < 0.001).

### 3.7 Overexpression of ATG7 reverses the inhibitory effect of MiR-582-5p on gastric cancer

**Figure 7:**
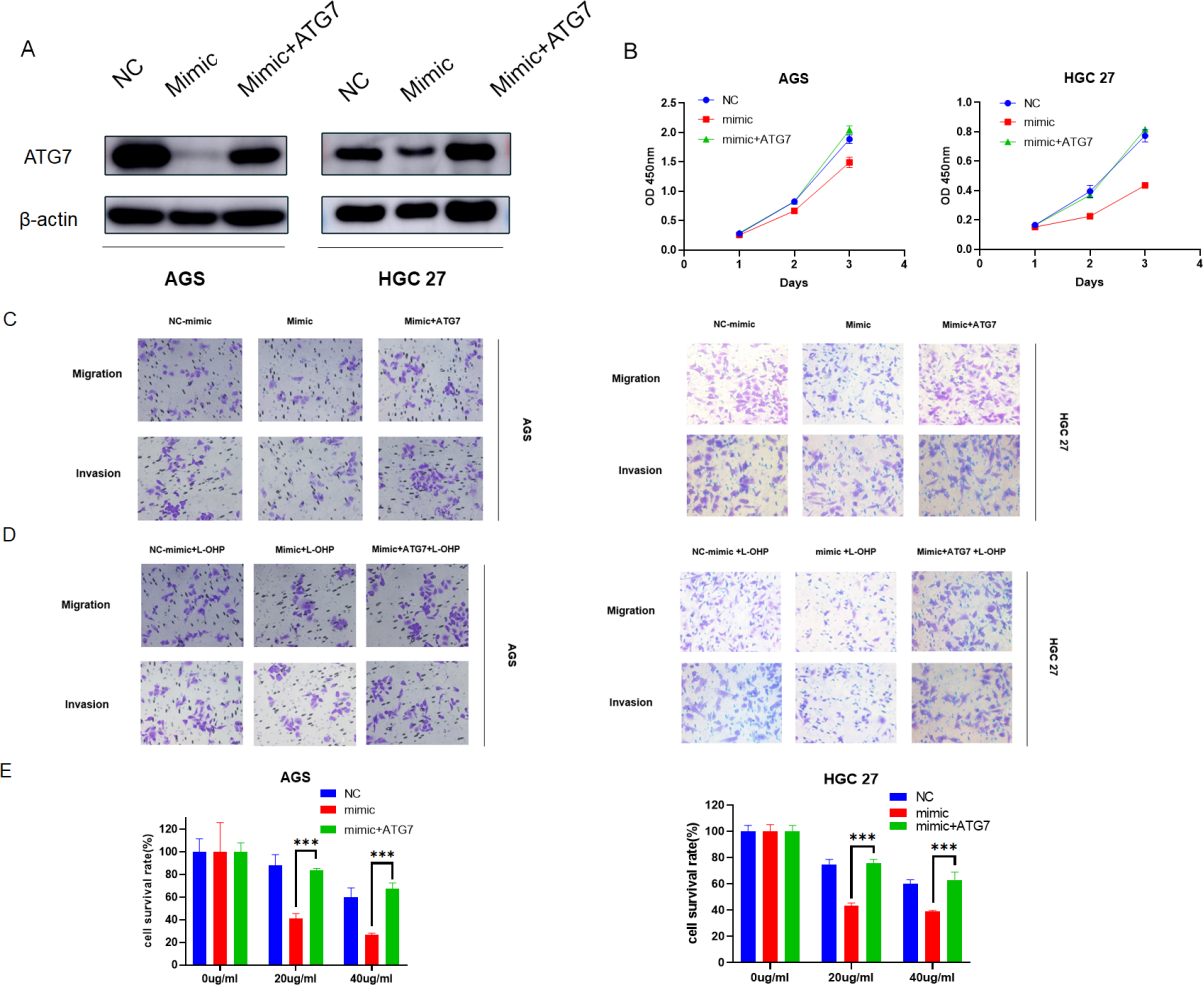
Overexpression of ATG7 reverses the inhibitory effect of MiR-582-5p on gastric cancer. **(A)** Expression levels of ATG7 in gastric cancer cells overexpressing MiR-582-5p alone and in combination with ATG7 overexpression. **(B)** Effects of overexpression of MiR-582-5p alone and in combination with ATG7 overexpression on the proliferative capacity of gastric cancer cells. **(C)** Effects of overexpression of MiR-582-5p alone and in combination with ATG7 overexpression on the migration and invasion abilities of gastric cancer cells. **(D, E)** Effects of overexpression of MiR-582-5p alone and in combination with ATG7 overexpression on the resistance of gastric cancer cells to oxaliplatin. (***P < 0.001).

To further demonstrate that MiR-582-5p exerts its effects by targeting ATG7, we conducted rescue experiments by co-overexpressing ATG7 and MiR-582-5p in gastric cancer cell lines. Western blotting confirmed that overexpression of MiR-582-5p reduced ATG7 expression, which was reversed upon simultaneous overexpression with ATG7 (**Figure 7A**). Subsequent functional assays and assessments of oxaliplatin sensitivity showed that co-overexpression of ATG7 reversed MiR-582-5p-induced inhibition of cell proliferation, as demonstrated by CCK8 assays (**Figure 7B**). Transwell assays further validated that co-overexpression of ATG7 reversed the phenotypic changes induced by MiR-582-5p overexpression in gastric cancer cells, including their sensitivity to oxaliplatin (**Figures 7C–E**).

## 4 Discussion

Gastric cancer is a malignant tumor originating from the epithelium of the stomach. The pathogenesis of gastric cancer is complex, influenced by various factors including gender, age, race, as well as modifiable factors such as Helicobacter pylori infection, obesity, unhealthy dietary habits, and lifestyle. Despite a general decline in the incidence and mortality rates of gastric cancer in recent years, it still ranks among the top five malignancies (22). Approximately 40%-60% of gastric cancer patients experience recurrence and metastasis, with two-thirds developing unresectable new lesions. The recurrence rate after surgery alone is high, with a 5-year overall survival (OS) of 23%-49%. Perioperative chemotherapy and adjuvant chemotherapy significantly improve patient survival and enhance quality of life (23). Chemotherapy has become a crucial treatment modality for gastric cancer patients. Current chemotherapy strategies include neoadjuvant triplet regimens like docetaxel, oxaliplatin, and S-1 (DOS), or epirubicin, cisplatin, and 5-fluorouracil (ECF), as well as doublet regimens such as capecitabine and oxaliplatin (XELOX), tegafur and cisplatin (SP), or 5-fluorouracil and oxaliplatin (FOLFOX). Despite significantly improving survival, chemotherapy tolerance and toxicity remain major challenges in the treatment of gastric cancer patients.

MicroRNAs have been demonstrated to play crucial roles in the development and progression of various cancers, particularly influencing cancer cell functions and sensitivity to treatment (24). MicroRNAs can act as oncogenes or tumor suppressors to regulate tumor cell growth (25). Their mechanisms of action include modulating the expression of target genes, acting as sponges for LncRNAs or CircRNAs, and participating in tumor-related signaling pathways. miR-582-5p has been found to modulate bladder cancer (18), colorectal cancer (19), prostate cancer (20), and non-small cell lung cancer (21). Recent studies have shown that LINC00641 targets miR-582-5p to activate autophagy levels in gastric cancer cells, thereby regulating their resistance to oxaliplatin (26). miR-582-5p can target AKT3 to inhibit proliferation of gastric cancer cells (27). However, whether miR-582-5p affects gastric cancer cell biological functions and sensitivity to oxaliplatin through targeting ATG7 has not been reported. Our study found that miR-582-5p is downregulated in gastric cancer and that overexpression of miR-582-5p inhibits proliferation, migration, invasion, and oxaliplatin resistance in gastric cancer cells. To further explore the potential mechanisms by which miR-582-5p exerts these functions, we validated the targeting relationship between miR-582-5p and ATG7, confirming the existence of potential binding sites.

ATG7 is an autophagy-related protein whose aberrant expression and its relationship with cancer are not yet fully understood. However, theoretically, inhibiting autophagy levels in cancer cells can suppress proliferation, migration, invasion, and resistance to anticancer treatments (11). Previous studies have demonstrated significant impacts of ATG7 on the invasive behavior of bladder cancer cells (28). Additionally, the long non-coding RNA NNT-AS1 regulates cisplatin resistance in lung cancer cells through the miR-1236-3p/ATG7 axis (29), indicating the critical role of ATG7-mediated autophagy in the biological functions and therapeutic resistance of malignant tumors.

Our current results indicate that ATG7 is a downstream target of miR-582-5p, and there is a negative correlation between ATG7 and miR-582-5p. miR-582-5p inhibits the expression of ATG7 in gastric cancer cells, thereby suppressing autophagy levels and influencing proliferation, migration, invasion, and resistance to oxaliplatin in gastric cancer cells.

In summary, we found that miR-582-5p is under-expressed in gastric cancer and can inhibit proliferation, migration, invasion, and oxaliplatin resistance of gastric cancer cells. Furthermore, we further confirmed that miR-582-5p suppresses cancer cell autophagy levels and epithelial-mesenchymal transition (EMT) by targeting ATG7 expression, thereby influencing the malignant biological functions and oxaliplatin resistance of gastric cancer cells.

## 5 Conclusion

We identified miR-582-5p as a novel tumor suppressor gene in gastric cancer. MiR-582-5p promotes invasion and metastasis of gastric cancer cells by negatively regulating ATG7 expression. We propose that miR-582-5p could serve as a potential therapeutic target in gastric cancer treatment.

## Data Availability

The original contributions presented in the study are included in the article/**Supplementary Material**. Further inquiries can be directed to the corresponding author.
