# Supplementary material for: MicroRNA-582-5p inhibits the progression of gastric cancer cells and their resistance to oxaliplatin by suppressing ATG7 expression
Source: Front Oncol. 2024 Oct 11;14:1481266. doi: 10.3389/fonc.2024.1481266 (PMC11502292; doi:10.3389/fonc.2024.1481266)
Supplement: Supplementary file 1 [file Table1.docx]

（1）qPCR primer sequences

| Title | primer sequences（5’to 3’） |
| --- | --- |
| Mir-582-5p | AGTGCAGGGTCCGAGGTATT  GCGTTACAGTTGTTCAACCAG |
| Mir-582-5p-RT | GTCGTATCCAGTGCAGGGTCCGAGGTATTCGCACTGGATACGACAGTAAC |
| ATG7 | CGCAGAGATGTGGAGCAACT  GCAGCAATGACGGCAGGA |
| GAPDH | GTCTCCTCTGACTTCAACAGCG  ACCACCCTGTTGCTGTAGCCAA |

（2）Si-ATG7 sequences

| Title | sequences（5’to 3’） |
| --- | --- |
| ATG7-si-1S | ACAUCAUUGCAGAAGUAGCAG |
| ATG7-si-1A | GCUACUUCUGCAAUGAUGUGG |
| ATG7-si-2S | AACAGAUACCAUCAAUUCCAC |
| ATG7-si-2A | GGAAUUGAUGGUAUCUGUUUU |

（3）Mimic and inhibitor sequences of Mir-582-5p

| Title | sequences（5’to 3’） |
| --- | --- |
| hsa-miR-582-5p | rUrUrArCrArGrUrUrGrUrUrCrArArCrCrArGrUrUrArCrU |
| hsa-miR-582-5p inhitor | rArG(2'-O-Me-U)rArA(2'-O-Me-C)(2'-O-Me-U)rGrG(2'-O-Me-U)  (2'-O-Me-U)rGrArA(2'-O-Me-C)rArA(2'-O-Me-C)(2'-O-Me-U)rG(2'-O-Me-U)rArA |

（4）Design of double luciferase report plasmid

| Title | sequences（5’to 3’） |
| --- | --- |
| ATG7 WT-hsa-mir-582-5p | CACCGCAGCTGACCCACTGCTCATCGCGAGGGCCTGCCAGGAGCTGGCCTCCCGC  ACTACTTGTGAGTAAAGTGAATATCAAATACCAATCTTAGAGTACAACTGTACCAGCA  GTAAGTATATCTAGGACTGTAACTGACAAAAATAAACTAATTCTGAAAAGAA |
| ATG7 MUT-hsa-mir-582-5p | CACCGCAGCTGACCCACTGCTCATCGCGAGGGCCTGCCAGGAGCTGGCCTCCCGCACTAC  TTGTGAGTAAAGTGAATATCAAATACCAATCTTAGAGTACCCAGTGCCCAGCAGTAAGTAT  ATCTAGGCAGTGCACTGACAAAAATAAACTAATTCTGAAAAGAA |
